# Peripheral Blood Mesenchymal Stem Cells and Platelet Rich Fibrin Matrix in the Management of Class II Gingival Recession: A Case Report

**DOI:** 10.30476/DENTJODS.2020.81784.0

**Published:** 2021-03

**Authors:** Sphoorthi Anup Belludi, Laveena Singhal, Madhuri Gubbala

**Affiliations:** 1 Dept. of Periodontics, K.L.E Society’s Institute of Dental Sciences, Bengaluru, Karnataka, India

**Keywords:** Peripheral blood mesenchymal stem cells, Platelet rich fibrin matrix, Coronally advanced flap, Gingival recession, Root coverage, Periodontal plastic procedure

## Abstract

The treatment of gingival recession is a frequent demand due to aesthetic concern, root caries, and /or root hypersensitivity. The purpose of this case study was to evaluate the success and predictability of coronally advanced flap (CAF) in combination with peripheral blood mesenchymal stem cells (PBMSCs) and platelet rich fibrin matrix(PRFM) for the management of Miller's Class II gingival recession. CAF followed by placement of PBMSCs and PRFM was performed on a male patient, aged 25 years having Miller’s Class II gingival recession of 5-6 mm on the upper left canine, premolars and molars. The patient was followed up for 3 months. Root coverage of 60.0% and clinical attachment gain of 3 mm were evident following 3 months of follow-up. This novel technique showed an effective way to increase the width of attached gingiva and treat gingival recession.

## Introduction

Gingival recession is defined as the displacement of the soft tissue margin apical to the cemento-enamel junction with exposure of root surface in the oral cavity [ 1 ]. It is one of the predominantly seen aesthetic concerns in periodontics. Therefore, obtaining predictable root coverage supported by a significant level of tissue regeneration has become a crucial aspect of periodontal plastic surgery.

Coronally advanced flap (CAF) when used alone, despite having advantages, is not reliable on a long-term basis [ 2 ]. Hence, such procedures do not assure the regeneration of lost attachment apparatus such as cementum, periodontal ligament, and alveolar bone, leading to a recurrence. Therefore, CAF is usually combined with a broad range of treatment alternatives like barrier membranes, autografts, demineralized freeze-dried bone allografts, bovine-derived xenografts, and combinations of membranes and fillers [ 3 ]. Until recently, only a few of these therapies have been considered as true regenerative techniques, regardless of their unpredictable outcomes. Autologous platelet concentrates represent promising innovative tools in periodontal regeneration field. Several studies have evaluated the effectiveness of platelet rich plasma (PRP) and platelet rich fibrin (PRF) in various clinical scenarios that require rapid healing and have found positive clinical and radiographic outcomes [ 4 - 6 ]. These positive outcomes have roused the need to extend and compare the usefulness of PRF to the newly developed platelet rich fibrin matrix (PRFM), which is a simplified process without artificial biomodification, representing a new step in the platelet gel therapeutic concept [ 7 ].

On the other hand, mesenchymal stem cells (MSCs) are multipotent stromal cell with prominent regenerative functions, which were for the first time identified and isolated from bone marrow. MSCs were later found in various tissues including peripheral blood, umbilical cord, adipose tissue and [ 8 ]. Lately increasing attention to peripheral blood mesenchymal stem cells (PBMSCs) is being drawn as they share identical biological properties with MSCs procured from either adipose tissue or bone marrow [ 9 ]. It would be very beneficial if PBMSCs could be procured and amplified to agreeable numbers, ensuring the osteogenic capacity is maintained in a clinically permitted period. Hence, the novel idea of combining these materials with their respective regenerative properties for treating Miller’s class II gingival recession to the best of our knowledge was done for the first time in the field of periodontics.

## Case Report

A 25-year-old male patient reported to the Department of Periodontology in KLE Society’s Institute of Dental Sciences, Bangalore with the chief complaint of receding gums and teeth sensitivity in maxillary left region. The patient noticed the presence of such an unaesthetic appearance 1 year back. On clinical examination, Miller’s class II gingival recession was noticed in relation to 23, 24, 25, 26 with shallow probing depth, mild bleeding on probing, thick gingival biotype and adequate width of attached gingiva. CAF with PRFM and PBMSCs was the choice of treatment to correct the recession defects. PRFM and PBMSCs (Supercell) was procured using a special kit (Meresis Supercell, DiponEd Biointelligence LLP, Bengaluru, KA, India), which consists of two separate tubes. The complete surgical procedure was explained in detail to the patient and written consent was obtained. Patient was advised to get a complete hemogram (blood investigations), which included total count (TC), differential count (DC), hemoglobin percent (Hb %), bleeding time (BT), clotting time (CT) and platelet count. Along with the complete hemogram, random blood sugar level (RBS) was also assessed. 

Scaling and root planing was carried out. The etiology of the recession was attributed to the patient’s history of forceful brushing with a hard brush. Hence, oral hygiene instructions also included the demonstration of brushing technique with adequate force and prescription of a soft brush in addition to other oral hygiene measures. Three weeks following the initial therapy, the periodontal re-evaluation was done. After re-evaluation, surgical procedure was carried out.

### Surgical procedure

**Figure 1:**
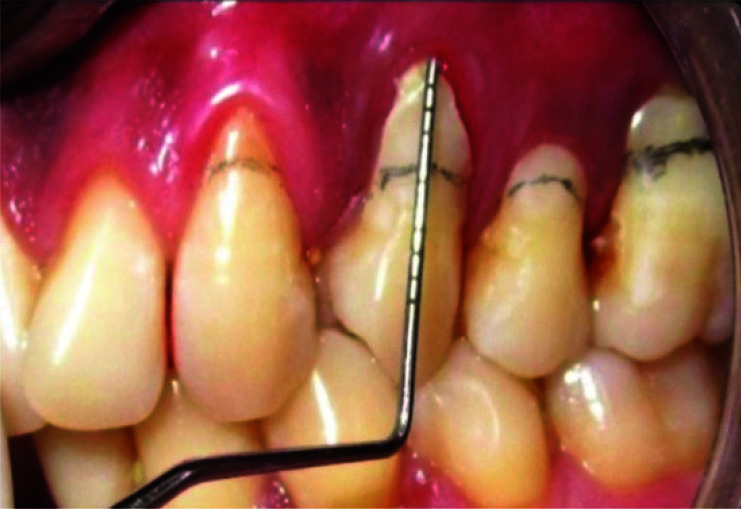
Preoperative analysis

**Figure 2:**
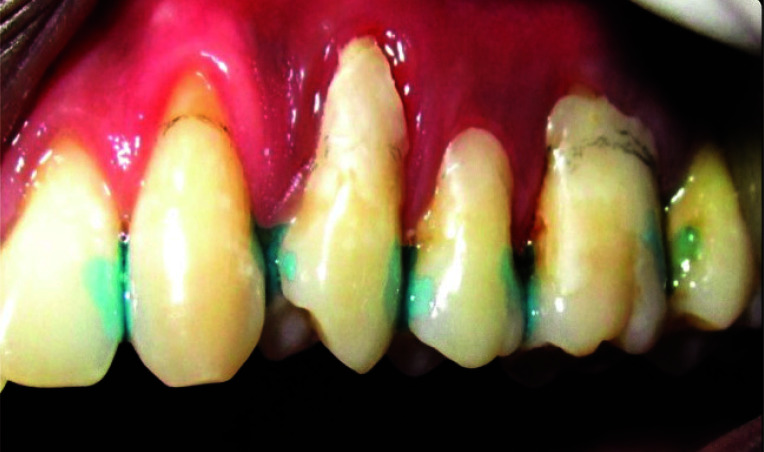
Blocking of the contact points with composite

**Figure 3:**
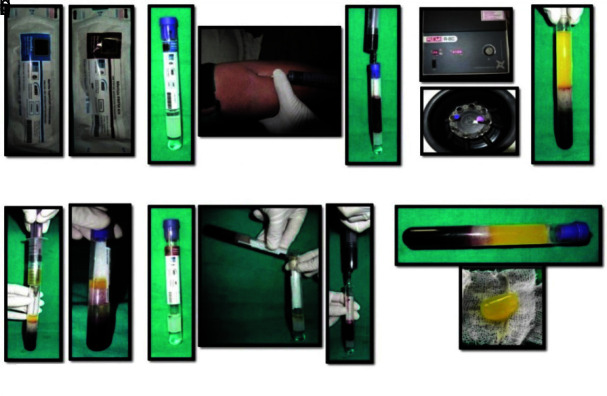
Preparation of supercell

**Figure 4:**
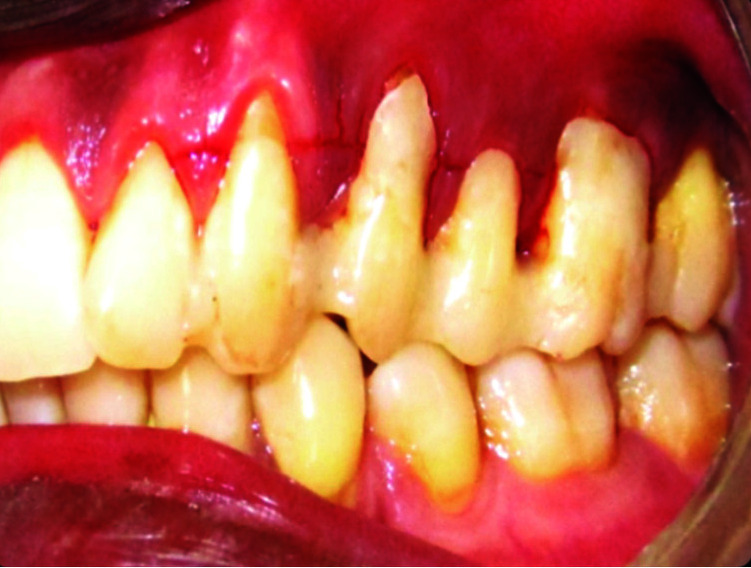
Anticipated root coverage marked

A preoperative analysis was made and the depth of the recession was evaluated, (Figure 1) followed by blocking the contact points with composite (Figure 2) to hold the sutures. The supercell was prepared following the protocol (Figure 3). First stem cells were prepared where 9 ml of patients’ blood was drawn into the Merisis supercell tube. The tube was then placed in a centrifuge (Remi R 8C, REMI, India) which has a swing bucket rotor .The tube was spun for 6 minutes at 3400 rpm to separate the blood into supernatant plasma and stem cell suspension. After centrifugation, RBCs were located below the cell separator gel and the stem cells i.e. 0.5-1 ml above the gel was kept aside for further use. 8 ml of patients’ blood was drawn again and mixed with 1 ml of stem cells prepared previously. The tube was then placed in the centrifuge and spun for 5 minutes at 3400 rpm to separate the blood into supernatant plasma and platelet suspension. After centrifugation, due to fibrin polymerization, supercell, which is the combination of stem cells and PRFM was formed and ready to use. Using sterile tweezers the fibrin clot was separated easily. It was placed in a sterile glass dish. Before use, the serum content was removed by slightly squeezing with the gauze piece. The anticipated root coverage was marked (Figure 4).

**Figure 5:**
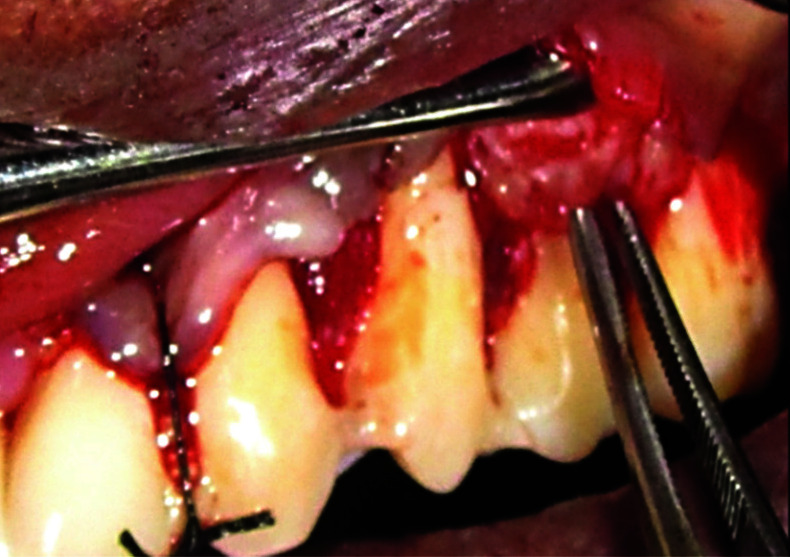
Placement of supercell

**Figure 6:**
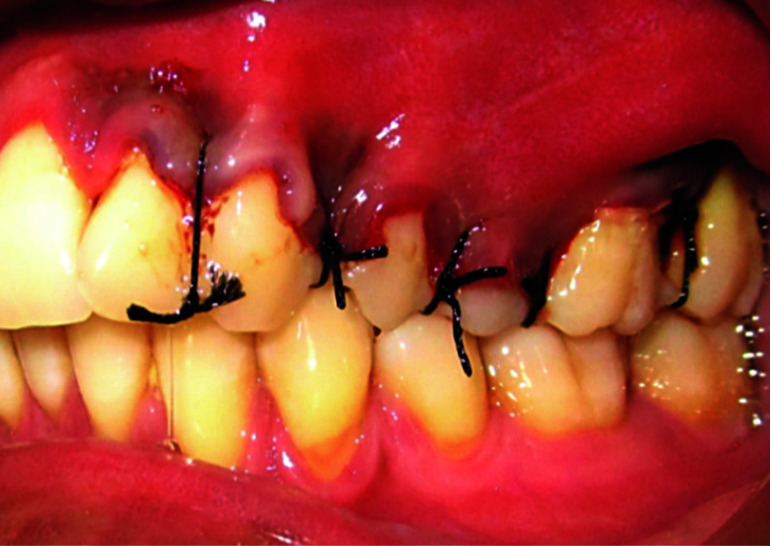
Coronal advancement of flap and placement of sutures

**Figure 7:**
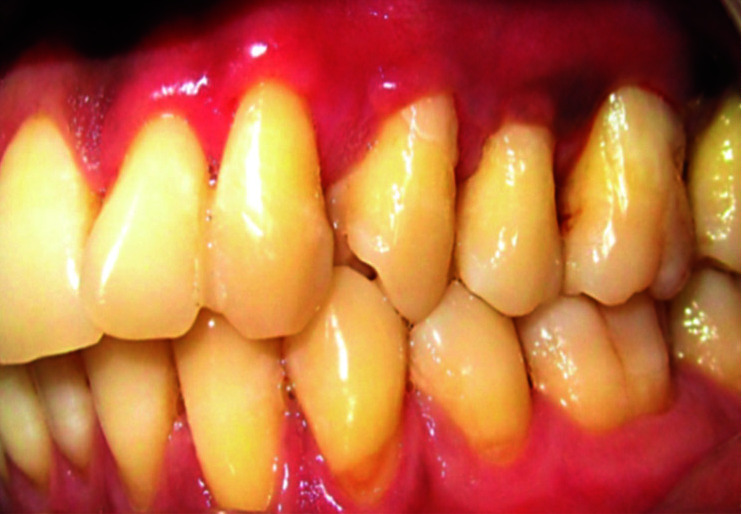
Three months after surgical procedure

After giving local anesthesia, initially, an intrasulcular incision extending from distal side of 22 to the distal side of 26 was given using blade number 15. A full thickness mucoperiosteal flap was elevated on the buccal aspect of the teeth being treated, followed apically with a partial thickness dissection beyond mucogingival junction. The area was debrided and cleaned thoroughly. Supercell was then placed (Figure 5) and flap was coronally advanced with its margin located on enamel. 4-0 non-resorbable silk sutures were used for suturing (Figure 6) followed by periodontal dressing. Patient was discharged with post-operative instructions and prescription and was recalled after 10 days for suture removal. Appreciable root coverage and soft tissue healing was noticed at that time. Three-month postoperative follow-up shows definitive root coverage (Figure 7).

## Discussion

The present case report aimed at treating multiple Miller’s Class-II gingival recessions with CAF and supercell. PRP, PRF, and platelet gels are autologous materials with high platelet concentration that integrate the advantageous properties of growth factors in platelets and fibrin sealants and thereby provide optimal growth factor release at the site of injury. The application of these preparations revolves around the tenet that, growth factors have a key role in soft and hard tissue repair mechanisms [ 10 ]. Amongst the various platelet concentrates, PRFM allows a sustained, effective dispensation of growth factors to the wound site.

They possess the ability to enhance tissue repair by isolation, concentration, and preservation of autologous platelets in a dense fibrin matrix [ 11 ]. Postnatal tissue-specific stem cells hold great promise in enhancing the repair of damaged tissues. Invasive procedures are required to procure most of these cells from either adipose tissue or bone marrow. Due to its ease of retrieval peripheral blood is a good substitutable source for stem cells or progenitor cells [ 12 ].

Clinical and histologic data reveal that the platelet concentrate therapeutic concept and recently stem cell technology would provide an encouraging medium for improvement of soft tissue healing and regeneration in periodontology. Accelerated periodontal healing and regenerative effects have popularized the indications of platelet concentrates in various surgical procedures in periodontics [ 13 ]. In the present case, report use of PRFM and PBMSCs along with CAF has shown good clinical outcomes and esthetic results. However, to assess the type of healing no histologic evaluation was performed. Therefore, the effect of PRFM and PBMSCs on the formation of a connective tissue attachment needs to be established. 

## Conclusion

The combination of PRFM and PBMSCs has a potential for both hard and soft tissue regeneration and is a promising material in the field of periodontal regeneration. This novel material has been used along with CAF procedure for the first time for root coverage, which has shown promising result. However, we can substantiate these results with further evaluation of this material through long-term follow-ups and by randomized controlled trials.
